# Capturing Amyloid-β Oligomers by Stirring with Microscaled Iron Oxide Stir Bars into Magnetic Plaques to Reduce Cytotoxicity toward Neuronal Cells

**DOI:** 10.3390/nano10071284

**Published:** 2020-06-30

**Authors:** Yuan-Chung Tsai, Jing-Chian Luo, Te-I Liu, I-Lin Lu, Ming-Yin Shen, Chun-Yu Chuang, Chorng-Shyan Chern, Hsin-Cheng Chiu

**Affiliations:** 1 Department of Biomedical Engineering and Environmental Sciences, National Tsing Hua University, Hsinchu 30013, Taiwan; smallqq_109@hotmail.com (Y.-C.T.); eric820713@hotmail.com (J.-C.L.); be0007599@gmail.com (T.-I.L.); b8201130@yahoo.com.tw (I.-L.L.); mingyin.shen@gmail.com (M.-Y.S.); cychuang@mx.nthu.edu.tw (C.-Y.C.); 2 Department of Surgery, Hsinchu Mackay Memorial Hospital, Hsinchu 30071, Taiwan; 3 Department of Surgery, China Medical University Hospital-Hsinchu Branch, Hsinchu 30059, Taiwan; 4 Department of Chemical Engineering, National Taiwan University of Science and Technology, Taipei 10607, Taiwan; cschern@mail.ntust.edu.tw

**Keywords:** Alzheimer’s disease, amyloid beta-peptides, microglial cell polarization, microscaled stirring, magnetic stir bars

## Abstract

Soluble amyloid-β oligomers (oAβ_42_)-induced neuronal death and inflammation response has been recognized as one of the major causes of Alzheimer’s disease (AD). In this work, a novel strategy adopting silica-coated iron oxide stir bar (MSB)-based AD therapy system via magnetic stirring-induced capture of oAβ_42_ into magnetic plaques (mpAβ_42_) and activation of microglia on cellular plaque clearance was developed. With oAβ_42_ being effectively converted into mpAβ_42_, the neurotoxicity toward neuronal cells was thus greatly reduced. In addition to the good preservation of neurite outgrowth through the diminished uptake of oAβ_42_, neurons treated with oAβ_42_ under magnetic stirring also exhibited comparable neuron-specific protein expression to those in the absence of oAβ_42_. The phagocytic uptake of mpAβ_42_ by microglia was enhanced significantly as compared to the counterpart of oAβ_42_, and the M1 polarization of microglia often occurring after the uptake of oAβ_42_ restricted to an appreciable extent. As a result, the inflammation induced by pro-inflammatory cytokines was greatly alleviated.

## 1. Introduction

The abnormal accumulation of amyloid-β oligomers (oAβ_42_) and protofibrils within neuronal/microglial cells is believed to be crucial to Alzheimer’s disease (AD) progression [1]. Although new strategies in therapeutic regimens including β-secreatase 1 (BACE1) inhibition, γ-secretase suppression, and passive anti-amyloid-β immunotherapies [2,3] have been proposed, the efficacy is still not significant probably due to the incomplete elimination of oAβ_42_ species in brain. For instance, an oral BACE1 inhibitor, Lanabecestat, was discontinued in phase III clinical study owing to its insignificant therapeutic efficacy [4]. A passive anti-amyloid-β antibody drug, solanezumab, was failed in clinical trial due to the poor improvement on cognitive impairment [5]. In addition to the neurotoxicity of oAβ_42_ from the impairment of synaptic plasticity in association with the interaction of oAβ_42_ with cellular prion protein [6,7], oAβ_42_ may further induce microglial cell polarization into M1 phenotype along with severe phagocytic function attenuation toward toxic species [8,9]. Moreover, the neuroinflammation induced by pro-inflammatory cytokines released from M1 type microglial cells further enhances the apoptosis of neuronal cells [10,11,12]. By contrast, the M2 phenotype microglial cells exhibit a superior phagocytic capability to eliminate Aβ_42_ in comparison with other resident central nervous system (CNS) immune cells such as M1 type microglial cells, astrocytes, and possibly neuronal cells [13,14,15]. Furthermore, the secretion of neurotrophic factors and anti-inflammatory cytokines from M2 microglial cells in brain regions helps repair CNS damage and alleviate inflammation [16]. Unfortunately, naive microglial cells localized in neuroinflammatory regions are apt to differentiate into the M1 phenotype, which further deteriorates inflammatory conditions [17,18]. 

It has been reported that sulindac sulfide and orcein-related drug (O) can be employed as stimulators for accelerating the aggregation of oAβ_42_, which reduce its neurotoxicity for effective AD treatment [19,20]. On the other hand, β-secretase 1 (BACE) inhibitor was found capable of reducing soluble form of Aβ_42_ (oAβ_42_) accompanied by rescue of cognitive dysfunction and memory deficits in a mouse model of AD [21]. Similarly, Meier et al. employed a BACE inhibitor (NB-360) to serve as a scavenger for Aβ reduction in tg-ArcSwe mice [22]. Nevertheless, these approaches are somewhat limited due to the ineffective elimination of oAβ_42_.

**Figure 1:**
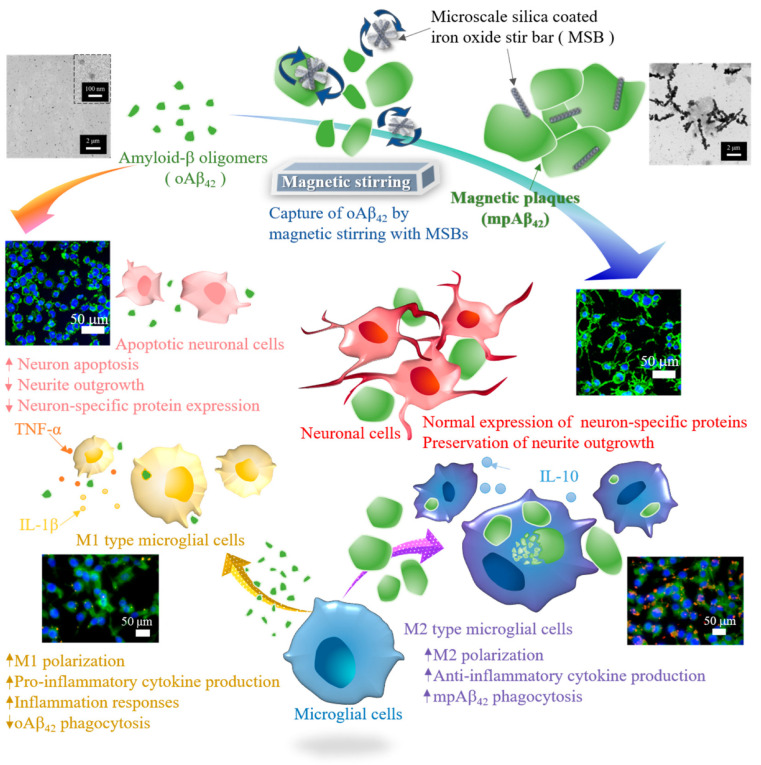
Illustration of the effective therapeutic action by capture of oAβ_42_ species into magnetic plaques and enhanced cellular clearance by the M2 type microglial cells for AD treatment.

This work was aimed to develop a novel therapeutic strategy to achieve rapid capture and subsequent removal of oAβ_42_ and prevention of neuroinflammation for potential AD treatment. Distinct from small molecule drugs as aforementioned that require a slow progression process involved in the oAβ_42_ aggregation, a MSB-based approach was developed and characterized in relation with the capability to effectively capture oAβ_42_ into mpAβ_42_ by facile magnetic stirring. The MSBs comprising aligned superparamagnetic iron oxide nanoparticles (SPIONs) coated by silica in rod shape were prepared and characterized with respect to the morphology by scanning electron microscopic (SEM) examination and the stirring effect under rotating magnetic field. The therapeutic effect on neuron cells by rapid capture of oAβ_42_ with the aid of MSBs under magnetic stirring into mpAβ_42_ in terms of cell viability and morphology, neuron-specific protein expression, and oAβ_42_-induced neuron membrane damage was extensively examined. A reduction in the uptake of oAβ_42_ by microglial cells also leads to a significant decrease in polarization of microglial cells into the M1 type. This will then greatly reduce the level of the pro-inflammatory cytokines. The internalization efficiency of oAβ_42_ and mpAβ_42_ by microglial cells and the effect on cell polarization was thus investigated. The in vivo biocompatibility of the MSB-based therapeutic system with stirring in terms of brain tissue damage from C57BL/6 mice was examined by the immunohistochemistry (IHC) staining. To the best of our knowledge, this is the first study demonstrating the rapid capture of oAβ_42_ by MSB under magnetic stirring and its potential for AD treatment. The therapeutic strategy developed herein is illustrated schematically in Figure 1.

## 2. Materials and Methods

### 2.1. Reagents and Materials 

Preparation of oleic acid coated SPIONs (OA-SPIONs) was conducted as reported previously [23]. Congo red (CR), thioflavin T (ThT), and hematoxylin and eosin (H&E) were purchased from Sigma-Aldrich (St. Louis, MO, USA). Tetraethyl orthosilicate (TEOS) and 1,1,1,3,3,3-hexafluoro-2-propanol (HFIP) were acquired from Alfa Aesar (Haverhill, MA, USA). Aβ_42_ peptide (DAEFRHDSGYEVHHQKLVFFAEDVGSNKGAIIGLMVGGVVIA) was acquired from Chinapeptide (Shanghai, China). Lactate dehydrogenase (LDH) assay kit, Hoechst 33342, rabbit anti-oligomer (A11) polyclonal antibody (AHB0052) and goat anti-mouse IgG secondary antibody (Alexa Fluor 568) were purchased from Thermo Fisher Scientific (Waltham, MA, USA). Anti-mouse F-actin antibody, goat anti-mouse IgG secondary antibody (Alexa Fluor 488), and donkey anti-rabbit IgG secondary antibody (Alexa Fluor 594) were obtained from Abcam (Cambridge, UK). Microtubule-associated protein 2 (MAP2) antibody and anti-human β-amyloid (6E10) antibody were purchased from BioLegend (San Diego, CA, USA). Neuronal nuclei (NeuN) antibody and neurofilament M (NF-M) antibody were supplied by Merck (Darmstadt, Germany). 4’,6-Diamidino-2-phenylindole (DAPI), singlet oxygen sensor green (SOSG) reagent and anti-mouse caspase-3 antibody were purchased by Invitrogen (Eugene, OR, USA). N2a (murine neuroblastoma cells) and BV-2 (murine microglial cells) were obtained from Food Industry Research and Development Institute (Hsinchu City, Taiwan). C57BL/6J male mice were purchased from the National Laboratory Animal Center, Taiwan. Approved guidelines for the care and use of laboratory animals by the Institutional Animal Care and Use Committee (IACUC: 10129) were followed. 

### 2.2. Preparation and Characterization of MSBs

The lauric acid/oleic acid coated SPIONs (LA/OA-SPIONs) were prepared using the oil-in-water emulsion technique. LA sodium salt (200 mg in 15 mL deionized water) and OA-SPIONs (15 mg in 200 μL chloroform) were mixed by ultrasonication for 10 min in an ice–water bath. This was followed by removal of chloroform by rotary evaporation. The resultant LA/OA-SPIONs were well dispersed in deionized water and phosphate buffered saline (PBS). The mean hydrodynamic diameters (D_h_), size distributions (polydispersity index, PDI), and zeta potentials of OA- and LA/OA-SPIONs in n-hexane and PBS, respectively, were determined by dynamic light scattering (DLS, ZetaSizer Nano Series, Malvern, UK). Preparation of MSBs was carried out according to the method previously reported [24]. The MSBs in microscale were prepared according to the method reported previously by neodymium magnet assisted alignment of SPIONs into rod-shaped structure in aqueous phase while the SPION surfaces were concomitantly coated with TEOS-based silica to maintain the desired particle morphology and improve the colloidal stability. In brief, LA/OA-SPIONs (0.05 mg) were dispersed in a mixture of isopropanol (1 mL) and deionized water (600 μL) under sonication. A NH_4_OH solution (28%, 20 μL) and TEOS (20 μL) was added in sequence into the aqueous suspension of LA/OA-SPIONs and the reaction solution was then placed between two 10 × 20 × 30 mm neodymium magnets (4,100 gauss) at a distance of 1.0 cm for 20 min at room temperature. After removal from the magnetic field, the sample was purified through centrifugation (1,900 g, 2 min) with deionized water three times and re-dispersed in deionized water. The morphology of SPIONs and MSBs was examined by transmission electron microscope (TEM) (T7700, Hitachi, Tokyo, Japan). SEM examination was conducted on a Hitachi S-4700 field emission microscope. Characterization of superparamagnetic properties was carried out on a superconducting quantum interference device (SQUID) magnetometer at 300 K (MPMS-XL Quantum Design, San Diego, CA, USA). The Fe content of OA-SPIONs was determined by thermogravimetric analyzer (TGA, Mettler-Toledo, Greifensee, Switzerland) at a heating rate of 10 °C min^−1^. X-ray diffraction (XRD) measurements of OA-SPIONs and LA/OA-SPIONs were conducted on a TTRax III diffractometer (Rigaku, Japan).

### 2.3. Preparation and Characterization of oAβ_42_ and Natural Aβ_42_ Plaques (npAβ_42_)

oAβ_42_ and npAβ_42_ were prepared and characterized according to the methods described previously [25,26,27,28]. The Aβ_42_ peptide was initially dissolved in HFIP (1.0 mM) and the solution was divided into aliquotes in sterile microcentrifuge tubes. HFIP was then removed under vacuum. The peptide film thus obtained was stored in dessicator at −20 °C before use. The β-sheet motif of npAβ_42_ was examined by ThT assay and CR spectroscopic analysis. For ThT assay, Aβ_42_ in dimethyl sulphoxide (DMSO) was added with 100 μL PBS (pH 7.4, ionic strength 0.15 M) to a concentration of 20 μM in 96-well plates. ThT was added (10 μM) and the solution was incubated at 37 °C as a function of time. The fluorescence intensity of ThT was determined by a microplate reader (Infinite M200 PRO, Männedorf, Switzerland). The excitation was performed at 442 nm and the emission spectrum was recorded in the range of 445–482 nm. The characteristic absorbance of CR at 490 nm and the red shift in the CR absorption band with the occurrence of npAβ_42_ in the range of 490–540 nm was determined using a UV–vis spectrophotometer. For the 100 μM oAβ_42_ preparation, the Aβ_42_ peptide film was first dissolved in dry DMSO to a concentration of 5 mM. The solution was then diluted with the Dulbecco’s Modified Eagle’s Medium (DMEM) (phenol red-free) to an Aβ_42_ concentration of 100 μM. The solution was further incubated at 4 °C for 24 h. The morphology of oAβ_42_ and npAβ_42_ was examined by transmission electron microscopy (TEM, HT7700, Hitachi, Tokyo, Japan). Briefly, the Aβ dispersion was dropped onto a carbon-coated copper grid (200 mesh copper grids, Ted Pella, Inc., Redding, CA, USA) and stained with 1 wt % uranyl acetate solution for 1 min and dried at 25 °C for 2 days before measurements. The stained samples were analyzed by TEM operating at an accelerating voltage of 100 kV.

### 2.4. In Vitro Stirring-Induced oAβ_42_ Capture

The capture effect of the magnetic stirring treatment with MSBs on oAβ_42_ species of varying concentrations (0.3–160 μM) under rotating magnetic field at 2500 rpm was determined by fluorescence microscope (Olympus IX70, Tokyo, Japan). Rotating magnetic stirring was conducted on an IKA magnetic stirrer (color squid, Staufen, Germany) with a dynamic magnetic attraction force in the range 15–30 mT. The stirring speed was calibrated with an external speed sensor, which reacts to magnetic impulses. The power consumption is 3 W and the motor rating output is 2 W. For the preparation of 20 μM oAβ_42_, the peptide film prepared previously was dissolved in dry DMSO to a concentration of 5 mM. The solution was then diluted with the DMEM medium (phenol red-free) to an Aβ_42_ concentration of 20 μM, followed by re-incubation at 4 °C for 24 h. The oAβ_42_ (20 μM) was first treated with magnetic stirring as a function of the concentration of MSBs at 2500 rpm for 20 min. The Aβ_42_ was then stained with 10 μM ThT (λex = 442 nm, λem = 445–482 nm) and CR (λex = 497 nm, λem = 596–632 nm) dyes. The total and mean areas of the stained mpAβ_42_ were quantified with the functions of Image-Pro Plus 6.0 (*n* = 10). The morphology of mpAβ_42_ was examined by TEM, scanning probe microscopy (SPM, tapping mode, Bruker, Model-Dimension Icon, Camarillo, CA, USA) and atomic force microscope (AFM, Bruker, Santa Barbara, CA, USA) [29,30,31,32].

### 2.5. Cell Membrane Damage and Neurite Outgrowth Impairment

3-(4,5-Dimethylthiazol-2-yl)-2,5-diphenyltetrazolium bromide (MTT) and trypan blue assays were used to determine the cell viability of N2a cells co-incubated with oAβ_42_ species (160 μM) under the MSB stirring treatment (2500 rpm, 2 h). For 160 μM oAβ_42_ preparation, the peptide film obtained previously was dissolved in dry DMSO to a concentration of 5 mM. The solution was then diluted with the DMEM medium (phenol red-free) to an Aβ_42_ concentration of 160 μM, followed by re-incubation at 4 °C for 24 h. N2a cells were first seeded into 96-well plates at a concentration of 5 × 10^3^ cells/well in 200 μL DMEM, containing 10% fetal bovine serum (FBS) and 1% penicillin, and incubated at 37 °C overnight in an atmosphere of 5% CO_2_. N2a cells were then co-incubated with oAβ_42_ (160 μM) with or without MSB-based (144 μg mL^−1^) stirring at 2500 rpm for another 2 h. After being washed twice with PBS, cells were re-incubated in DMEM for another 24 h. MTT (0.25 μg/mL) in DMEM (200 μL) was added into each well, followed by re-incubation at 37 °C for 4 h. The culture medium was then replaced with DMSO (100 μL) and the absorbance of each well at 570 nm was determined by the microplate reader. The MTT assay for the cell viability evaluation was performed based upon the measurement of the cellular metabolic activity, in particular with respect to the activity of mitochondria reductase (dehydrogenase) by colorimetric determination. Trypan blue assay was used to determine the number of viable N2a cells. In brief, trypan blue solution (0.4%) in DMEM (200 μL) was co-incubated with N2a cells being treated with either oAβ_42_ (160 μM) alone or oAβ_42_/magnetic stirring (2500 rpm, 2 h) for 24 h at 37 °C. The viable N2a cells with intact cell membranes that prevent trypan blue from permeation into cells was then examined by optical microscopy. The effects of oAβ_42_ and mpAβ_42_ on neuron apoptosis in terms of the level of cell membrane damage was evaluated by measuring the release of LDH, a cytosolic enzyme from the damage cells [33]. The neurite outgrowth was examined by laser scanning confocal microscope (LSCM, ZEISS LSM-780, Jena, Germany). The cell nuclei and cytoskeletons were stained with Hoechst 33342 and CytoPainter F-actin Staining Kit-Green Fluorescence, respectively. Mean number and length (μm) of neurites were calculated by Neurolucida software (Version 8, MBF Bioscience, USA), respectively. The mean neurite length was defined herein as the ratio of the sum of neurite length to the number of neurite. At least three independent experiments were performed in this study, and at least 50 cells quantified per experiment.

### 2.6. Cell Functions

The cellular functions in terms of specific protein expression were examined by western blotting. Proteins were subjected to a 12% sodium dodecyl sulfate-polyacrylamide gel electrophoresis (SDS-PAGE) and transferred onto polyvinylidene difluoride (PVDF) membrane. The PVDF membrane was blocked using 5% nonfat milk blocking buffer with anti-MAP-2, NF-M, and NeuN primary antibody at 4 °C for 24 h. It was then incubated at 37 °C for 1 h with horseradish peroxidase-conjugated secondary antibody. β-Actin was used as the loading control. The area and signal intensity of the detected bands were determined using the Molecular Imager PharosFX Plus System and quantified using the ImageJ software (v1.33, National Institute of Mental Health, MD, USA). For the LSCM examination, N2a cells on glass coverslips were treated with MSBs (144 μg), free oAβ_42_ (160 μM), and oAβ_42_/MSBs, respectively, either with or without 2 h magnetic stirring (2500 rpm) at 37 °C. With additional 22 h incubation, the cells were washed with PBS three times and fixed with 4% formaldehyde. Triton X-100 (0.1%) was added to increase the cell permeability. After being rinsed with PBS, the cells were then co-incubated in sequence with mouse anti-mouse MAP-2 antibody as the primary antibody and goat anti-mouse Alexa Fluor 488 as the secondary antibody at ambient temperature. Cells were further IHC stained for NF-M similarly while the rabbit anti mouse NF-M and donkey anti-rabbit antibodies (conjugated with Alexa Fluor 594) as primary and secondary antibody was employed for NF-M detection. Finally, cells were stained with Hoechst 33342 (5 μg mL^−1^) to identify cell nuclei.

### 2.7. Phagocytic Activity of Microglial Cells

BV-2 cells were adopted as a microglia cell model for evaluating the uptake of Aβ_42_ in different forms (i.e., oAβ_42_, npAβ_42_ and mpAβ_42_). BV-2 cells (3 × 10^5^ cells per well) were seeded on glass coverslips and cultured at 37 °C under 5% CO_2_/air overnight. This was followed by co-incubation with the Aβ_42_ species at varying concentrations. With 6 h co-incubation, the coverslips were washed with PBS twice, fixed with 4% paraformaldehyde and stained with 6E10 for 10 min in sequence. The extent of cellular uptake of Aβ_42_ in different forms by microglia was visualized at the excitation and emission wavelengths of 488 nm and 590 nm, respectively, with LSCM.

### 2.8. Cytokine Production

Levels of IL-1β, TNF-α and IL-10 released from BV-2 cells after uptake of Aβ_42_ species of various structures were examined by enzyme-linked immunosorbent assay (ELISA). A 96-well tailor-made mouse multi-analyte ELISArray kit (Qiagen, SA Biosciences, Hilden, UK) was employed to determine the concentrations of TNF-α, IL-1β, and IL-10 in the collected culture media according to the manufacturer’s instruction.

### 2.9. Brain Tissue Toxicity and Damage with MSB Stirring

The intracranial injection of 2 μL of the aqueous MSB dispersion (0.5 and 1.0 μg in 2 mL of F-12K medium, respectively) into the hippocampus area of healthy C57BL/6J mice with the magnetic stirring was conducted in this work to evaluate the in vivo brain tissue toxicity and damage by MSB stirring [34,35,36]. In brief, male C57BL/6J mice (6–8 weeks old) were first anesthetized with Zoletil-Rompun by intraperitoneal injection and placed in an animal stereotaxic apparatus (Stoelting, Wood Dale, IL, USA). Two μL of the aqueous MSB dispersion were injected intracranially into the hippocampus area using a 10 μL Hamilton syringe equipped on the stereotaxic instrument with a 27-gauge needle and a microinjection auto-pump (KD Scientific, Hollison, MA, USA). After the needle was gently withdrawn, the burr hole was immediately sealed with bone wax and the surgical incision was sutured. This was followed by magnetic stirring (2400 rpm) over 20 min. At the prescribed time points, the brains were isolated and sectioned. The reactive oxygen species (ROS) level induced from Fe^2+^ and Fe^3+^ ions (Fenton reaction) with the dissolution of MSBs was determined by SOSG. The cell apoptosis in brain tissue sections was examined by caspase 3 IHC staining. The Prussian blue staining was used to determine the Fe content in brain tissue with time. The hippocampus tissue sections were H&E stained for histologic examination under optical microscopy.

### 2.10. Statistical Analysis

All experiments were performed at least in triplicate and data were reported as mean ± standard deviation. Statistical significance was determined using one-way ANOVA and Bonferroni post-hoc test. Significant differences were defined as (*) *p* < 0.05, (**) *p* < 0.01, and (***) *p* < 0.005. Not significant differences were displayed as N.S. (*p* > 0.05).

## 3. Results and Discussion

### 3.1. Preparation and Characterization of MSBs

**Figure 2:**
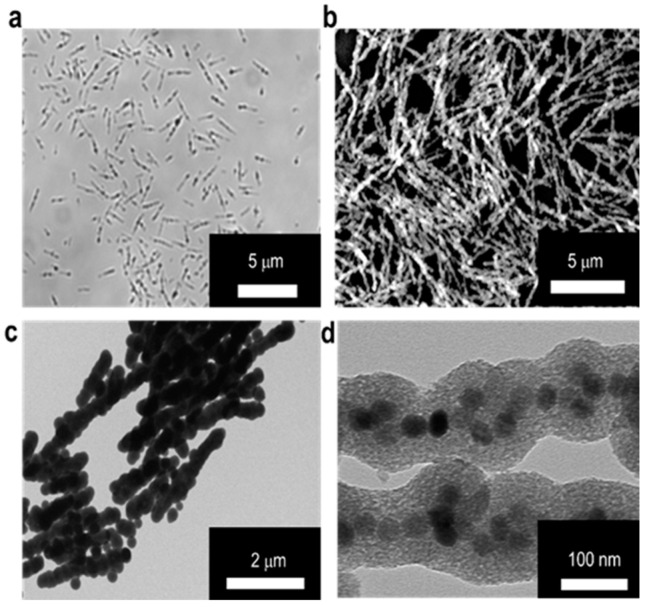
Characterization of microscale silica-coated iron oxide stir bars (MSBs). Morphology of MSBs examined by (**a**) optical microscope in aqueous solution and (**b**) SEM in dry state. (**c**) TEM image of MSBs. Scale bar: 2 μm. (**d**) HR-TEM image of MSBs. Scale bar: 100 nm.

The MSBs in microscale were prepared according to the method reported previously [24]. Through the neodymium magnet assisted alignment of SPIONs into rod-shaped structure in aqueous phase, the SPION surfaces were concomitantly coated with TEOS-based silica to maintain the desired particle morphology and improve the colloidal stability. The particle surface of SPIONs in the form of OA-SPIONs prepared by the thermal decomposition technique were modified with LA by hydrophobic association to improve the aqueous colloidal dispersion. The results obtained from characterization of OA-SPIONs and LA/OA-SPIONs by TEM, TGA, SQUID, and XRD are shown in Figure S1. TEM images of LA/OA-SPIONs and OA-SPIONs confirm their spherical particle morphology. The resultant LA/OA-SPIONs still retain excellent superparamagnetic behavior with similar hysteresis to OA-SPIONs (Figure S1d). In addition, the XRD patterns of OA-SPIONs and LA/OA-SPIONs show high crystallinity in the spherical phase structure of Fe_3_O_4_ (JCPDS: 65-3107) (Figure S1e). Table S1 also shows the mean hydrodynamic diameters of OA-SPIONs in n-hexane and LA/OA-SPIONs in PBS and the zeta potential of LA/OA-SPIONs at pH 7.4. The optical microscopic, SEM, TEM, and high resolution TEM (HR-TEM) images of the MSBs developed herein are shown in Figure 2. The dimensions of MSBs were ca. 2.9 ± 0.7 μm in length and 132.4 ± 4.1 nm in diameter with the thickness of silica coating at ca. 45.5 ± 5.2 nm estimated by TEM image. The HR-TEM image further confirms the confinement of LA/OA-SPIONs in a relatively linear architecture within the silica coat (Figure 2d).

In the absence of magnetic field, MSBs remained as rod-like particles randomly dispersed in the continuous aqueous phase, as illustrated in Figure S2a. By contrast, MSBs became uniformly aligned in response to the direction of the applied external magnetic field (Figure S2b). The stirring effect of MSBs was observed by the accelerated dispersion of rhodamine B employed as a fluorescence dye in a water droplet in the presence of MSBs under an external magnetic rotating field (Figure S2c). Without magnetic stirring, the dye species resided locally within the water droplet. On the contrary, the dye species subjected to magnetic stirring became rapidly dispersed in the droplet and the extent of dispersion was dependent on the stirring speed and duration. Notably, a homogenous aqueous solution of rhodamine B was achieved over 20 s of stirring at 2500 rpm. The rapid rotation of MSBs is illustrated in Video S1, in which individual rotations of MSBs at various stirring speeds were recorded. The blinking phenomenon caused by light scattering during magnetic stirring was also observed (Video S2), similar to what has been reported elsewhere [37]. The colloidal stability of MSBs in different aqueous milieus was also evaluated in terms of the time-evolved change of light scattering intensity by DLS measurements. Figure S3 illustrates negligible variations in count rate of the aqueous MSB dispersions over a period of 1 h, indicating excellent colloidal stability under 500; 1500; or 2500 rpm magnetic stirring. Similar observation from the silica-coated nanoparticles was reported in the literature [38]. Being quite similar in surface modification, silica coating exhibited a strong resistance against both salt-induced aggregation in PBS and protein adsorption-evolved coagulation in Dulbecco’s modified Eagle medium (DMEM) solution primarily because of the high negative surface charge density of MSBs (ζ-potential of ca. −35 mV).

### 3.2. Capture Efficiency of Aβ_42_ by Magnetic Stirring of MSBs and In Vitro Therapeutic Efficacy

**Figure 3:**
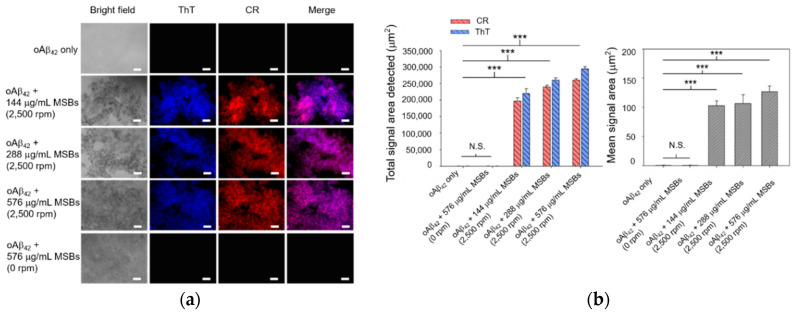
Capture of oAβ_42_ into mpAβ_42_ by magnetic stirring with MSBs. (**a**) Fluorescence images of oAβ_42_ (20 μM) treated with magnetic stirring as a function of the concentration of MSBs at 2500 rpm for 20 min (*n* = 10). Scale bar: 100 μm. (**b**) The total and mean signal areas detected from the Aβ_42_ aggregation induced by magnetic stirring with MSBs were included. *** *p* < 0.005 and N.S. *p* > 0.05. Error bars represent mean ± s.d. (*n* = 10).

Rapid agglutination and capture of oAβ_42_ into mpAβ_42_ in cell culture medium by means of magnetic stirring with MSBs was studied first by fluorescence microscopy. As shown by the fluorescence images in Figure 3, the signal areas of both ThT and CR in the aqueous oAβ_42_ solution subjected to magnetic stirring treatment increase dramatically compared to those without magnetic stirring treatment. These signals were observed obviously owing to the formation of oAβ_42_ aggregates. The total areas detected are in the range 2 × 10^5^~3 × 10^5^ μm^2^ while the mean areas in the range 110–130 μm^2^ with oAβ_42_ being treated with magnetic stirring (Figure 3). The signal areas from ThT and CR overlapped mostly, indicating that both staining occurred at the same places. At constant stirring speed (2500 rpm), a rather weak dependence of the total signal area of the ThT/CR stained Aβ_42_ aggregates on the concentration of MSB in the range 144–576 μg/mL employed herein was observed. The effect of the MSB concentration (144–576 μg/mL) on the mean signal area (i.e., the oAβ_42_ aggregate size) is also essentially insignificant. In contrast to the magnetic stirring effect, the fluorescence signals of both ThT and CR were barely detected from the aqueous solution of oAβ_42_ without stirring over a period of 20 min. This result strongly suggests that the oAβ_42_ aggregate formation is substantially promoted by the magnetic stirring provided by MSBs as compared to the counterpart that is not subjected to stirring. It is noteworthy that the sound capture of oAβ_42_ is successfully achieved by magnetic stirring even at a concentration of 0.3 μM. This implies the potential of the magnetic stirring with MSBs in practical applications for AD treatment (Figure S4).

**Figure 4:**
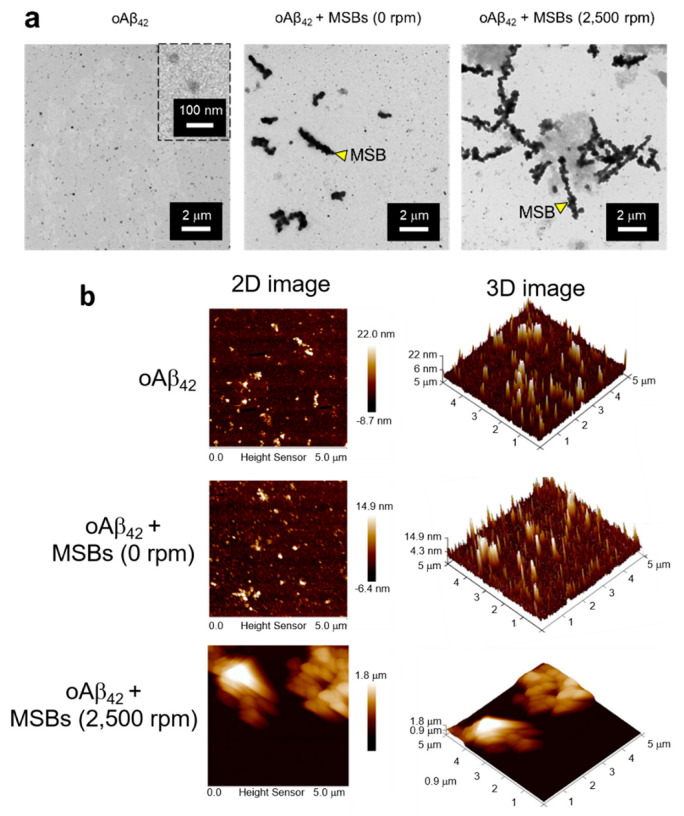
Structural analyses of oAβ_42_ and mpAβ_42_ by TEM and AFM. (**a**) TEM images of effective capture of oAβ_42_ into mpAβ_42_ via magnetic stirring. Scale bar: 2 μm. (**b**) Examination of mpAβ_42_ by AFM. 2D topographic images of oAβ_42_, oAβ_42_ + MSBs (0 rpm), and oAβ_42_ + MSBs (2500 rpm); and 3D topographic representations of oAβ_42_, oAβ_42_ + MSBs (0 rpm), and oAβ_42_ + MSBs (2500 rpm).

The capture of oAβ_42_ into mpAβ_42_ via magnetic stirring with MSBs was further supported by comparison of the oAβ_42_ images before and after the magnetic stirring treatment using the TEM and AFM techniques (Figure 4). The TEM images clearly demonstrate the agglutination of oAβ_42_ after the magnetic stirring with MSBs while the small oAβ_42_ species were well separated from MSBs in the absence of the magnetic stirring treatment (Figure 4a). Both the 2-D and 3-D AFM topographic maps also corroborate the oAβ_42_ plaque formation promoted by MSB stirring with the plaque size being enormously enlarged in comparison with the oligomers (Figure 4b).

Figure S5a shows that the fluorescence intensity of ThT in the aqueous oAβ_42_ solution increases significantly as the time-evolved npAβ_42_ form although over a relatively prolonged period of time (5–7 days) is required. The increase of the fluorescence intensity is ascribed to the restriction of the rotation of both benzylamine and benzathiole rings of ThT once the dye binds with Aβ_42_ in the forms of both fibrils and naturally occurring plaques which are enriched with the β-sheet structure [39,40,41,42]. Similar to the data reported elsewhere [43,44], a red shift in the wavelength of the maximum absorbance of CR from 490 to 540 nm was observed upon the association of CR with the insoluble β-sheet rich Aβ_42_ aggregates such as fibrils and npAβ_42_ (Figure S5b). Figure S5c,d show the TEM images of oAβ_42_ and npAβ_42_, respectively. The latter was obtained from the incubation of oAβ_42_ in PBS at 37 °C under mild shaking over 7 days. TEM images show that npAβ_42_ fully comprises fibrils while the aggregate size (ca. 70 μm^2^ attained from the fluorescence microscopic examination) is somewhat smaller than that of mpAβ_42_ (ca. 125 μm^2^). It is interesting to note that the ThT fluorescence intensity reaches a plateau value of ca. 35,000 (a.u.) for the npAβ_42_ formation compared to ca. 20,000 for ThT with mpAβ_42_ at the same concentration of Aβ_42_ (20 μM) and ca. 1000 for ThT alone in aqueous solution. Comparing the structures of npAβ_42_ and oAβ_42_ shown by TEM images, obviously oAβ_42_ species are rather void of the fibril structure (Figure S5c), which by contrast predominates in npAβ_42_ (Figure S5d). With mpAβ_42_ being formed via the rapid capture of oAβ_42_ with the MSB stirring treatment, a decrease in the fluorescence intensity of ThT when bound with mpAβ_42_ in comparison with the signal intensity associated with the npAβ_42_ formation can thus be expected. Since the size of npAβ_42_ is smaller than that of mpAβ_42_, the effect of the magnetic stirring treatment on npAβ_42_ was also examined. Figure S6a shows the capability of the MSB stirring to readily capture npAβ_42_ (20 μM) into larger aggregates at a speed of 2500 rpm over 20 min. The extent of aggregation in terms of the detected total signal area closely correlating to the concentration of MSB employed for magnetic stirring was also observed (Figure S6b).

**Figure 5:**
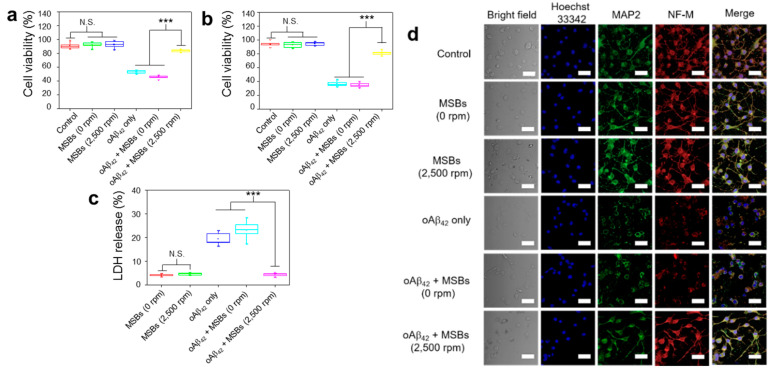
Effective therapeutic action by capture of oAβ_42_ under magnetic stirring with MSBs. (**a**) In vitro cytotoxicity of N2a cells with or without magnetic stirring treatment by MTT and (**b**) trypan blue assays. The experiment was independently repeated three times. Error bars show standard deviations. (**c**) LDH release from N2a cells with and without the oAβ_42_ capture by the MSB stirring treatment. (**d**) Effects of oAβ_42_ on neuron-specific protein expression (MAP2 and NF-M) with and without the magnetic stirring treatment (*n* = 6). Cell nuclei were stained with Hoechst 33342 (λ_ex_ = 405 nm, λ_em_ = 450–500 nm), microtubules with MAP2 marker (λ_ex_ = 495 nm, λ_em_ = 500–550 nm) and neurofilaments with NF-M marker (λ_ex_ = 633 nm, λ_em_ = 640–660 nm). Scale bar: 50 μm. *** *p* < 0.005 and N.S. *p* > 0.05. Error bars represent mean ± s.d. (*n* = 8).

The effects of structural transformation from oAβ_42_ to mpAβ_42_ by magnetic stirring treatment on neuron cells (N2a) was investigated in terms of cell viability, cell morphology, and cell-specific protein expression. First, the in vitro cytotoxicity of mpAβ_42_ induced by the rapid capture of oAβ_42_ species against N2a cells was evaluated using MTT and trypan blue assays. In the absence of oAβ_42_, MSBs (144 μg mL^−1^) without or with magnetic stirring at 2500 rpm for 2 h resulted in negligible cell death as measured by MTT assay (Figure 5a). The cell viability was also barely affected by the stirring speed (0; 500; 1500; and 2500 rpm) and the concentration of MSB (up to 144 μg mL^−1^) employed for the rapid oAβ_42_ capture in the culture medium (Figure S7). By contrast, with those cells being exposed to oAβ_42_ without the stirring treatment, the cell viability was significantly reduced compared to the cell only control. This result is consistent with those reported previously that oAβ_42_ species are responsible in large measure for neuron toxicity in AD [45,46,47,48]. Nevertheless, an essentially full retention of the viability of N2a cells incubated with oAβ_42_ was observed when the N2a/oAβ_42_ suspension was concomitantly treated with the MSB stirring. The greatly reduced cytotoxicity is ascribed to the rapid capture of the oAβ_42_ into large aggregates by which the Aβ_42_ structure is appreciably altered and their entry into N2a cells extremely limited. The cytotoxicity of oAβ_42_ against N2a cells via the MSB stirring treatment was also evaluated by the trypan blue staining as a measure of cell apoptosis. As shown in Figure 5b, the cytotoxicity of oAβ_42_ was appreciably reduced as these toxic species were captured and converted into large magnetic aggregates. Figure 5c illustrates that the LDH release from N2a cells caused by oAβ_42_ is greatly reduced by the magnetic stirring treatment in comparison with the run without the treatment. Apparently, the cytotoxicity of oAβ_42_ species was considerably alleviated by their magnetic capture into large aggregates, thereby leading to the significant reduction of cellular uptake of oAβ_42_ by N2a cells for neurotoxicity attenuation. Figure 5d demonstrates the comparable neuron-specific protein expression of N2a cells in the presence of oAβ_42_ subjected to magnetic stirring to the positive control. In contrast, the neuron specific protein expression was significantly suppressed for the run without the capture of oAβ_42_ species into large aggregates of oAβ_42_/MSBs. Note that the proteins chosen for study include MAP2 and NF-M. Finally, further supporting evidence was provided by the neurite morphology. As shown in Figure S8, the morphology of N2a cells, in particular the neurites (axons and dendrites), was well preserved when they were treated by oAβ_42_ in virtue of the superior capture effect of magnetic stirring. The neurite length of N2a cells (ca. 20 μm) in the magnetic stirring-treated group was well retained as comparable to the positive control without oAβ_42_. By contrast, the length of neurites was reduced to ca. 3 μm with the cells being treated with oAβ_42_ only. All these results strongly suggest that the MSB stirring treatment is highly capable of capturing toxic oAβ_42_ species and, consequently, reducing their toxicity against neuronal cells. As a result, rather high cell integrity and viability are achieved.

### 3.3. Functionality of N2a Cells and Phagocytic Action of BV-2 Cells

**Figure 6:**
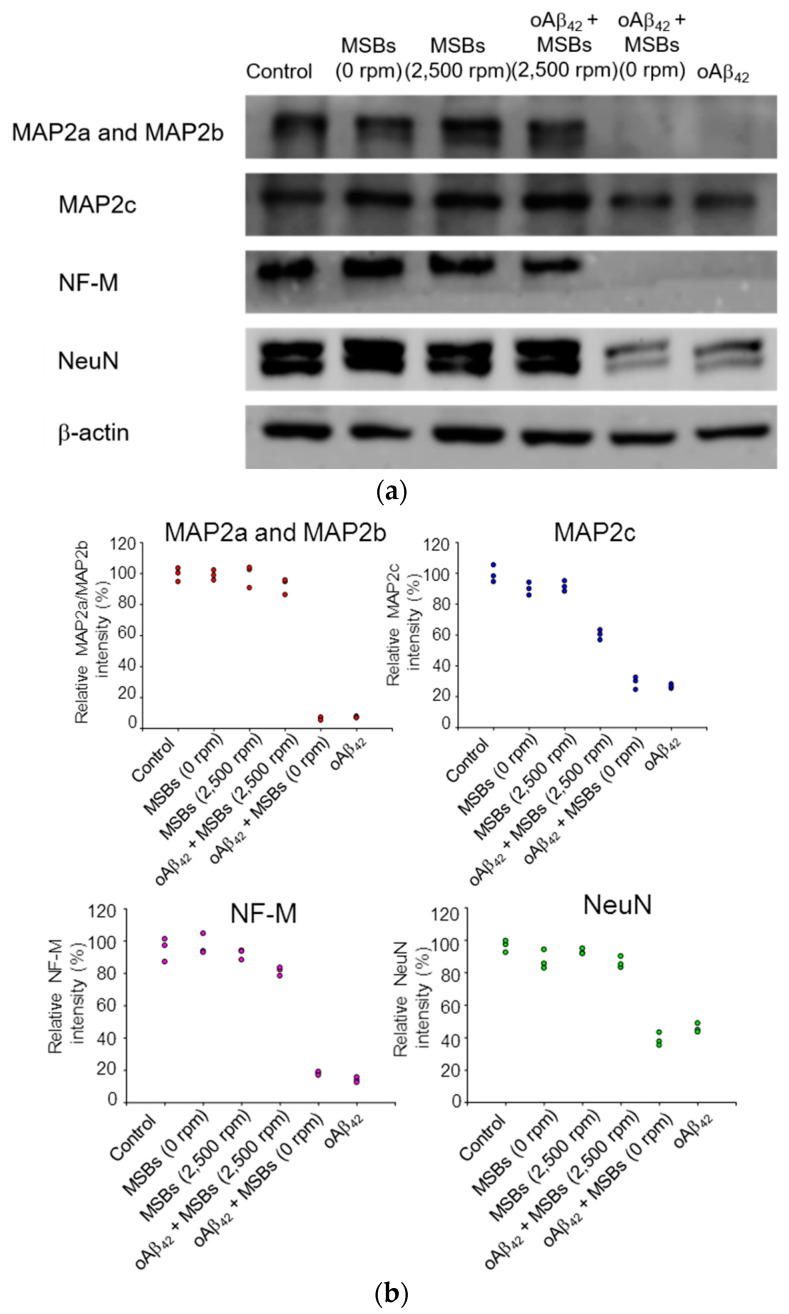
Neuron-specific protein expression of N2a cells receiving the magnetic stirring treatment. (**a**) Effects of oAβ_42_ on protein expression (MAP2a/MAP2b, MAP2c, NF-M, and NeuN) with and without the magnetic stirring (*n* = 3). β-actin was used as the loading control. The oAβ_42_ concentration was 160 μM. (**b**) The relative intensities of neuron-specific protein expression of N2a cells receiving magnetic stirring with MSBs were included.

The cellular functions in terms of specific protein expression after the rapid oAβ_42_ capture treatment on neuron cells were examined by western blotting. Figure 6 demonstrates that the MAP2 proteins, the NF-M protein, and NeuN were found normally expressed from N2a cells with a negligible effect of oAβ_42_ under magnetic stirring. On the other hand, the specific protein expression was significantly reduced in the negative control by oAβ_42_ without magnetic stirring. The quantification of the specific protein expression is also shown in Figure 6. Moreover, in the presence of npAβ_42_ instead of oAβ_42_, the specific expression of MAP2a, MAP2b, MAP2c, or NF-M on N2a cells was comparable to the blank control. This signifies that the neurotoxicity of both npAβ_42_ and mpAβ_42_ against N2a cells is substantially reduced compared to oAβ_42_ (Figure S9). In excellent agreement with the data obtained from the cell viability and morphology (Figure 5 and Figure S8), these results strongly imply that the effective therapeutic action is indeed achieved by capture of oAβ_42_ via MSBs under rotating magnetic field.

**Figure 7:**
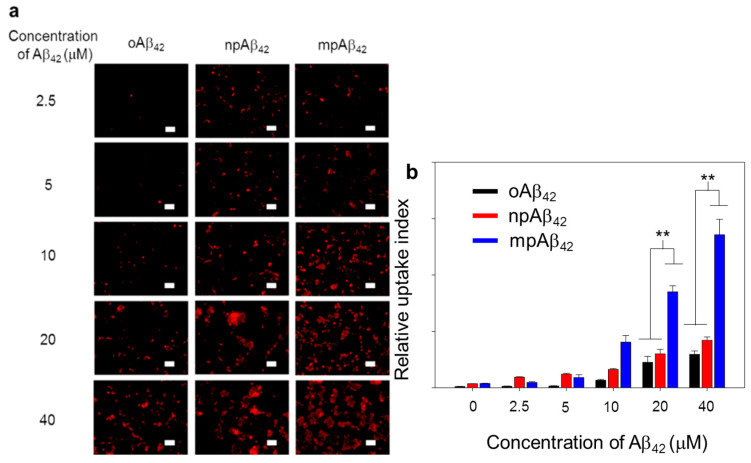
Phagocytic action of BV-2 cells. (**a**) Laser scanning confocal microscope (LSCM) images of BV-2 cells after co-incubation with oAβ_42_, npAβ_42_, and mpAβ_42_, respectively (*n* = 8). Aβ_42_ in different forms was IHC stained using 6E10 as the primary antibody (λ_ex_ = 565 nm, λ_em_ = 680–730 nm). Scale bar: 50 μm. (**b**) Relative uptake index of Aβ_42_ by BV-2 cells. One-way ANOVA was used to examine the mean differences between the end points of the data groups. ** *p* < 0.01. Error bars represent mean ± s.d. (*n* = 8).

As illustrated in Figure 7 and Figure S10, BV-2 cells (as a microglial cell model) treated with oAβ_42_ species exhibited quite weak fluorescence signals from Triton X-100 facilitated intracellular IHC staining using 6E10 as the primary antibody. In contrast, the fluorescence signal intensity was greatly enhanced for the cells treated with either npAβ_42_ or mpAβ_42_. This is most likely due to the enhanced uptake of mpAβ_42_ (or npAβ_42_) aggregates with activating the toll-like receptor (TLR)-associated endocytosis [49,50,51,52]. It was reported that the interactions of TLR-4 with CD14 on cell membranes were enhanced through the activation of CD14 upon their binding with Aβ_42_ species, triggering the TLR/CD14-mediated endocytosis [53]. On the other hand, the enhanced uptake of mpAβ_42_ by BV-2 cells compared to npAβ_42_ (Figure 7) is presumably caused by their enlarged size that inevitably increases contacts with CD14 on the cell membrane, thereby facilitating the TLR-mediated phagocytosis. It has been recognized that the internalization of oAβ_42_ species by microglia induces the M1 phenotype polarization of the cells, known for the reduced phagocytic activity toward neurotoxic species [54]. This will then lead to an increase in neuron apoptosis.

**Figure 8:**
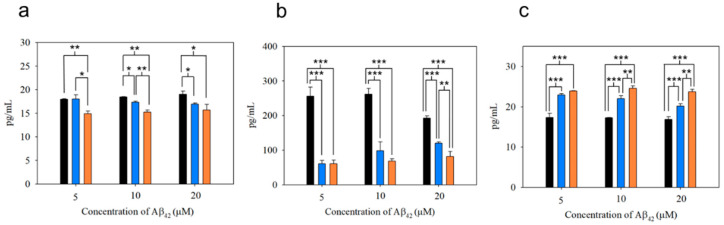
Cytokine secretion from BV-2 cells treated with oAβ_42_, mpAβ_42_, and npAβ_42_, respectively, for 6 h. Concentrations of IL-1β (**a**), TNF-α (**b**), and IL-10 (**c**) were determined in triplicate wells by ELISA analyses. The experiments were independently repeated three times and one-way ANOVA was used to examine the mean differences between the data groups. *** *p* < 0.005, ** *p* < 0.01, * *p* < 0.05, and N.S. *p* > 0.05. Error bars represent mean ± s.d. (*n* = 3).

As shown in Figure 8a,b, the production of the pro-inflammatory cytokines such as IL-1β and TNF-α from microglial cells after uptake of mpAβ_42_ (or npAβ_42_) was substantially reduced as compared to the uptake of oAβ_42_. This is also reflected by a significant increase in secretion of IL-10 (an anti-inflammatory cytokine) from microglial cells co-incubated with mpAβ_42_ (or npAβ_42_) aggregates as compared to oAβ_42_ species (Figure 8c). Comparing the differences in the levels of both pro-inflammatory cytokines and anti-inflammatory cytokines from microglia treated with oAβ_42_ species and plaques (mpAβ_42_ and npAβ_42_), respectively, the extent of the oAβ_42_-induced polarization of microglia to M1 type was reduced for the run with oAβ_42_ being captured and transformed into mpAβ_42_ (or npAβ_42_) via the magnetic stirring treatment. With the decreased pro-inflammatory cytokines and the increased anti-inflammatory cytokines, the neuroinflammation as one of the AD causes is thus greatly reduced. As shown in Figure S11, a significant difference in the viability of N2a cells after 24 h incubation in conditioned media collected separately from the co-incubations of BV-2 cells with oAβ_42_ and those with mpAβ_42_ was observed. Owing to the reduced inflammatory responses, the viability of the neuron cells in conditioned medium from the co-incubation of BV-2 cells with the stirring-induced Aβ_42_ aggregates was thus promoted. These data strongly imply that the magnetic stirring treatment is capable of effectively reducing neuroinflammation, thereby leading to a pronounced increase in the survival of N2a cells. In addition, the preliminary in vivo study showed no obvious tissue damage with the brain receiving the MSB stirring treatment (Figure S12a). The MSBs were mostly degraded over 15 days after the intrahippocampal injection (Figure S12b). No significant increases in cell apoptosis and ROS generation were found from the staining of the brain tissues with caspase 3 marker and with SOSG in the treated mice compared to the control group (Figure S12c). These results further demonstrated the safety and feasibility of this novel approach in practical applications for the AD treatment.

## 4. Conclusions

In this work, we have demonstrated the successful development of the MSB-stirring based AD therapy system for rapid effective capture of oAβ_42_ species that are subsequently transformed into large magnetic aggregates (mpAβ_42_) in vitro. In this manner, the neurotoxicity of oAβ_42_ against neuronal cells and the oAβ_42_-induced polarization of microglia to M1 type characterized by high pro-inflammatory propensity can be significantly reduced. Through the increased close contact among oAβ_42_ species by stirring and magnetic attraction of MSB-entrapped oAβ_42_ aggregates under rotating magnetic field, rapid effective conversion of neurotoxic oAβ_42_ species into harmless mpAβ_42_ can be achieved. The MSB therapy system not only exhibits negligible cytotoxicity toward neuronal cells in the absence or presence of rotating magnetic field, but also greatly reduces the neurotoxicity of oAβ_42_ by rapid capture of oAβ_42_ species into mpAβ_42_ aggregates even with a concentration at 0.3 μM. The magnetic stirring treatment has shown a superior capability of maintaining the functionality of N2a cells in viability, specific protein expression and neurite outgrowth, and reducing the levels of pro-inflammatory cytokines secreted from M1 type microglial cells due to the effective agglutination of toxic oAβ_42_ species into mpAβ_42_. With the limited polarization of microglia into M1 type, the effective clearance of mpAβ_42_ via the phagocytosis of microglia was achieved. The brain tissue histologic study showed no obvious tissue damage with the mice receiving the MSB stirring treatment in the hippocampus area. Neither cell apoptosis nor ROS generation in the brain tissues were found to be significantly increased. The MSBs were extensively degraded over 15 days after the intracranial injection. These results strongly suggest that the magnetic stirring treatment with MSBs be a promising strategy for improving the AD treatment. Further detailed studies concerning the in vivo therapeutic efficacy of the MSB stirring treatment against AD is in progress.

## Supplementary Materials

The following are available online at https://www.mdpi.com/2079-4991/10/7/1284/s1. Figure S1: Characterization of SPIONs. (a) TEM image of OA-SPIONs and (b) TEM image of LA/OA-SPIONs. Scale bar: 50 nm. (c) TGA measurement of OA-SPIONs. (d) Field-dependent magnetic curves of OA-SPIONs (black line) and LA/OA-SPIONs (red line) measured at the same Fe concentration. (e) XRD patterns of OA-SPIONs and LA/OA-SPIONs. Figure S2: Alignment of MSBs examined by optical microscopy. (a) The random localization of MSBs in the absence of external magnet. (b) Uniform alignment of MSBs in accordance to the magnet (black arrows). (c) Dispersion of rhodamine B in water droplets by magnetic stirring with MSBs under rotating magnetic field. Figure S3: Colloidal stability over 120 min in different aqueous milieus. Count rates of MSBs in DI water (━), DMEM (━), DMEM containing 10% FBS (━) and PBS (━) at different time intervals with various magnetic stirring speeds: (a) 0, (b) 500, (c) 1500 and (d) 2500 rpm. Error bars represent mean ± s.d. (*n* = 6). Figure S4: Pronounced effect of capturing oAβ_42_ by magnetic stirring (2500 rpm) with MSBs (144 μg/mL) into aggregates was observed by LSCM (*n* = 10). The oAβ_42_ was stained with ThT and CR. Scale bar: 100 μm. Figure S5: Detailed structural characterizations of npAβ_42_. (a) Fluorescence intensity of ThT in aqueous solution of Aβ_42_ (20 μM) as a function of time. (b) CR absorption spectra (420–580 nm) of Aβ_42_ (20 μM) with different incubation time intervals. (c) TEM and HR-TEM images of oAβ_42_. (d) TEM and HR-TEM images of npAβ_42_. Figure S6: Capture of npAβ_42_ into large aggregates by MSB stirring (*n* = 10). (a) Fluorescence images of npAβ_42_ treated with magnetic stirring as a function of the concentration of MSBs at 2500 rpm for 20 min. The Aβ_42_ concentration was 20 μM. The npAβ_42_ was attained from incubation of oAβ_42_ in PBS under mild shaking at 37 °C for 7 days. Scale bar: 100 μm. (b) Total signal areas of large aggregates by ThT and CR staining. *** *p* < 0.005, ** *p* < 0.01, * *p* < 0.05 and N.S. *p* > 0.05. Error bars represent mean ± s.d. (*n* = 10). Figure S7: (a) LSCM images of the neuron (N2a) outgrowth with magnetic stirring of various speeds for 2 h (MSB 144 μg mL^−1^). Cytoskeleton was stained with F-actin marker. Scale bar: 100 μm. The viability of N2a cells receiving the magnetic stirring treatment with MSBs (144 μg mL^−1^) at various stirring speeds. (b) Cell viability of N2a cells after the capture of oAβ_42_ by magnetic stirring with MSBs of different concentrations at 2500 rpm for preset time intervals. The cell viability was evaluated by MTT assay. Error bars represent mean ± s.d. (*n* = 6). Figure S8: Representative LSCM images of N2a cells before and after the magnetic stirring treatment. LSCM images of N2a cells treated with either oAβ_42_ only or oAβ_42_/MSBs (oAβ_42_ concentration: 160 μM; MSB 144 μg mL^−1^) with and without magnetic stirring (2500 rpm) for 2 h (*n* = 6). The cell nuclei and cytoskeleton were stained with Hoechst 33342 and F-actin marker, respectively. Scale bar: 50 μm. Quantitative data of neurite length (μm) and number with and without magnetic stirring treatment are also included. *** *p* < 0.005. Error bars represent mean ± s.d. (*n* = 6). Figure S9: Neuron-specific protein expression of N2a cells treated either with npAβ_42_ or mpAβ_42_ (*n* = 3). (a) Effects of npAβ_42_ and mpAβ_42_ on neuron-specific protein expression (MAP2a/MAP2b, MAP2c, NF-M and NeuN). β-actin was used as the loading control. (b) Relative signal intensities of individual neuron-specific proteins from N2a cells after the npAβ_42_ or mpAβ_42_ treatment. The Aβ_42_ concentration was 160 μM. * *p* < 0.05. Error bars represent mean ± s.d. (*n* = 3). Figure S10: Phagocytic actions of BV-2 cells toward oAβ_42_, npAβ_42_ and mpAβ_42_. Representative LSCM images of the Aβ_42_ uptake by BV-2 cells (*n* = 8). Aβ_42_ in different forms was IHC-stained using 6E10 as the primary antibody (λex = 565 nm, λem = 680–730 nm). Cell nuclei and cytoskeleton were stained with Hoechst 33342 and F-actin marker, respectively. Scale bar: 50 μm. Figure S11: Cell viability of N2a cells after 24 h-incubation in conditioned media collected separately from the co-incubations of BV-2 cells with oAβ_42_ and BV-2 cells with mpAβ_42_. *** *p* < 0.005, ** *p* < 0.01. Error bars represent mean ± s.d. (*n* = 6). Figure S12: Brain tissue examination with (a) H&E (b) prussian blue (MSBs) and (c) DAPI (nuclei), SOSG (ROS) and caspase-3 (cell apoptosis) IHC staining from healthy C57BL/6J mice receiving MSB stirring treatment (2400 rpm, 20 min) (*n* = 3). Scale bars for (a) and (b): 500 μm. Scale bar for (c): 100 μm. Table S1: Characterization of OA-SPIONs and LA/OA-SPIONs by DLS. Video S1: Magnetic sitrring of MSBs recorded by charged-coupled device (CCD) video camera. Vidoe S2: The blinking phenomenon caused by light scattering with MSB stirring recorded by charged-coupled device (CCD) video camera.
