# Supplementary material for: Capturing Amyloid-β Oligomers by Stirring with Microscaled Iron Oxide Stir Bars into Magnetic Plaques to Reduce Cytotoxicity toward Neuronal Cells
Source: Nanomaterials (Basel). 2020 Jun 30;10(7):1284. doi: 10.3390/nano10071284 (PMC7407479; doi:10.3390/nano10071284)
Supplement: Supplementary file 1 [file nanomaterials-10-01284-s001.zip › supplementary/Supplementary Materials.pdf]

Supplementary Materials

Capturing amyloid-β oligomers by stirring with microscaled iron oxide stir bars into magnetic plaques to reduce cytotoxicity toward neuronal cells

Yuan-Chung Tsai, Jing-Chian Luo, Te-I Liu, I-Lin Lu, Ming-Yin Shen, Chun-Yu Chuang, Chorng-Shyan Chern and Hsin-Cheng Chiu*

Dr. Yuan-Chung Tsai, Mr. Jing-Chian Luo, Dr. Te-I Liu, Dr. I-Lin Lu, Dr. Ming-Yin Shen, Prof. Chun-Yu Chuang, and Prof. Hsin-Cheng Chiu
Department of Biomedical Engineering and Environmental Sciences, National Tsing Hua University, Hsinchu 30013, Taiwan
E-mail addresses: hscchiu@mx.nthu.edu.tw (H.-C. Chiu)
Dr. I-Lin Lu
Department of Surgery, Hsinchu Mackay Memorial Hospital, Hsinchu 30071, Taiwan
Dr. Ming-Yin
Department of Surgery, China Medical University Hospital-Hsinchu Branch, Hsinchu 30059, Taiwan
Prof. Chorng-Shyan Chern
Department of Chemical Engineering, National Taiwan University of Science and Technology, Taipei 10607, Taiwan

Supplementary Figures……………..……………………………………………….……..03
Supplementary Table………………………………………………………...………16

Supplementary Figures

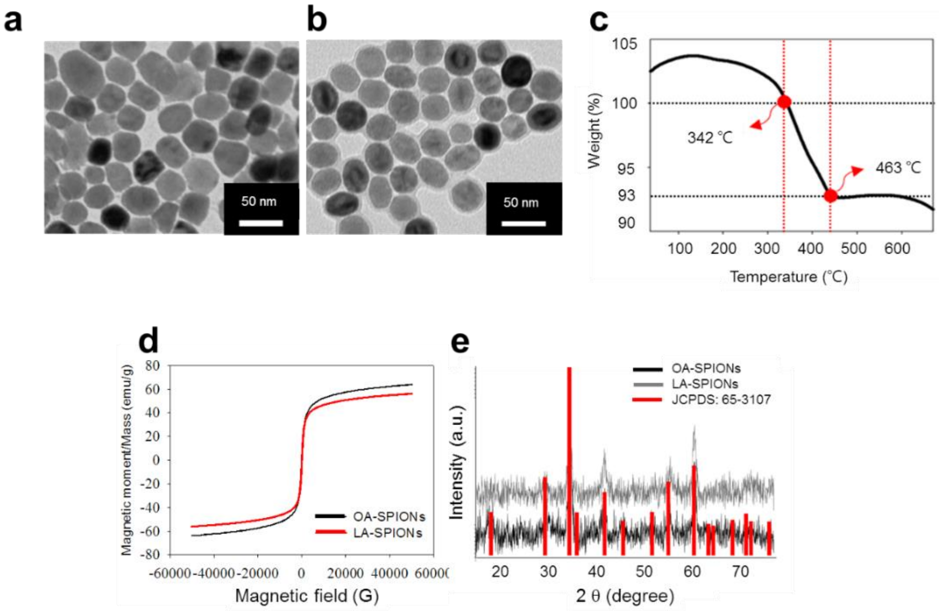

Fig. S1 Characterization of SPIONs. (**a**) TEM image of OA-SPIONs and (**b**) TEM image of LA/OA-SPIONs. Scale bar: 50 nm. (**c**) TGA measurement of OA-SPIONs. (**d**) Field-dependent magnetic curves of OA-SPIONs (black line) and LA/OA-SPIONs (red line) measured at the same Fe concentration. (**e**) XRD patterns of OA-SPIONs and LA/OA-SPIONs.

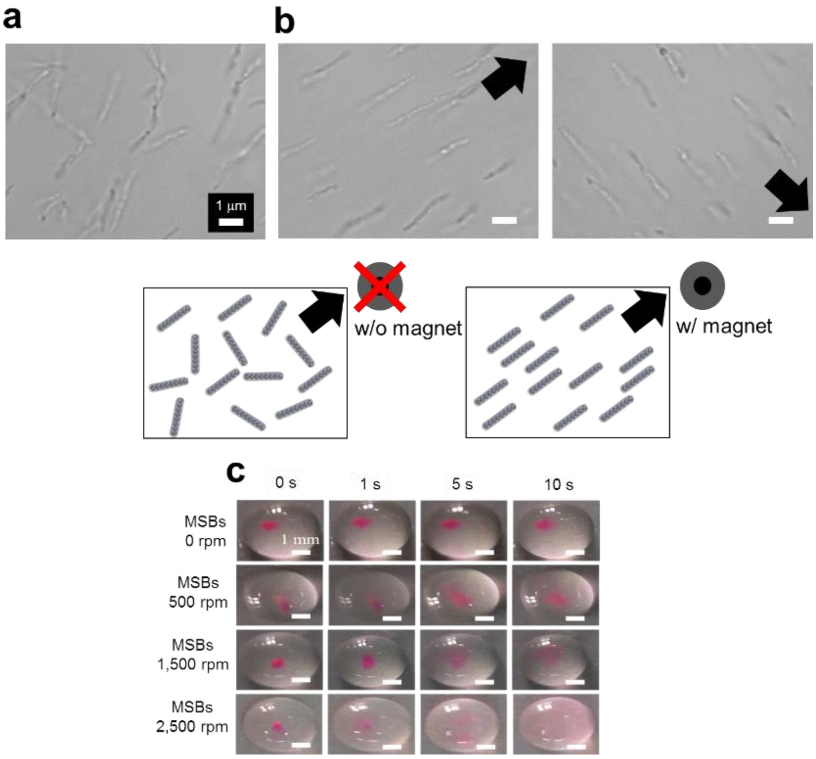

Fig. S2 Alignment of MSBs examined by optical microscopy. (**a**) The random localization of MSBs in the absence of external magnet. (**b**) Uniform alignment of MSBs in accordance to the magnet (black arrows). (**c**) Dispersion of rhodamine B in water droplets by magnetic stirring with MSBs under rotating magnetic field.

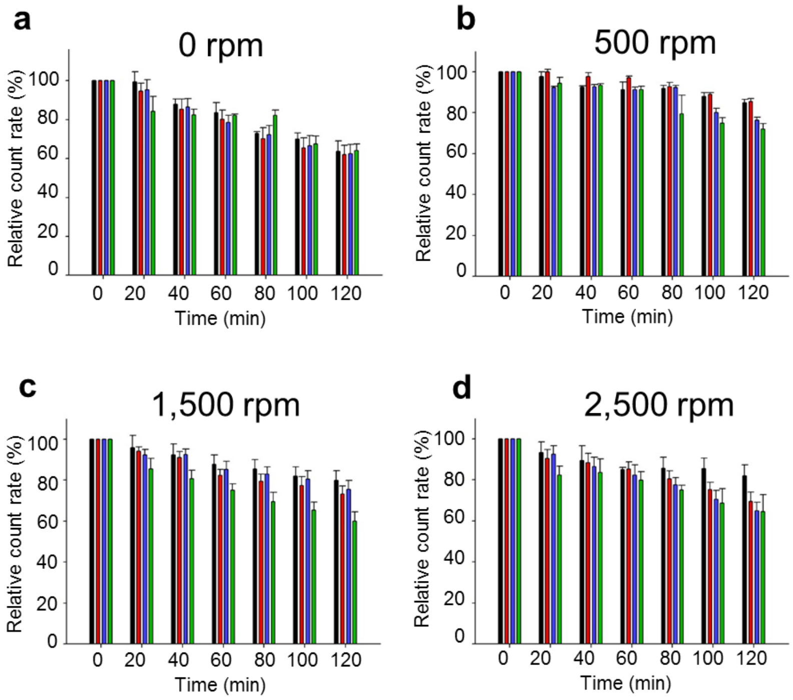

Fig. S3 Colloidal stability over 120 min in different aqueous milieus. Count rates of MSBs in DI water (—), DMEM (—), DMEM containing 10% FBS (—) and PBS (—) at different time intervals with various magnetic stirring speeds: (**a**) 0, (**b**) 500, (**c**) 1,500 and (**d**) 2,500 rpm. Error bars represent mean ± s.d. (n = 6).

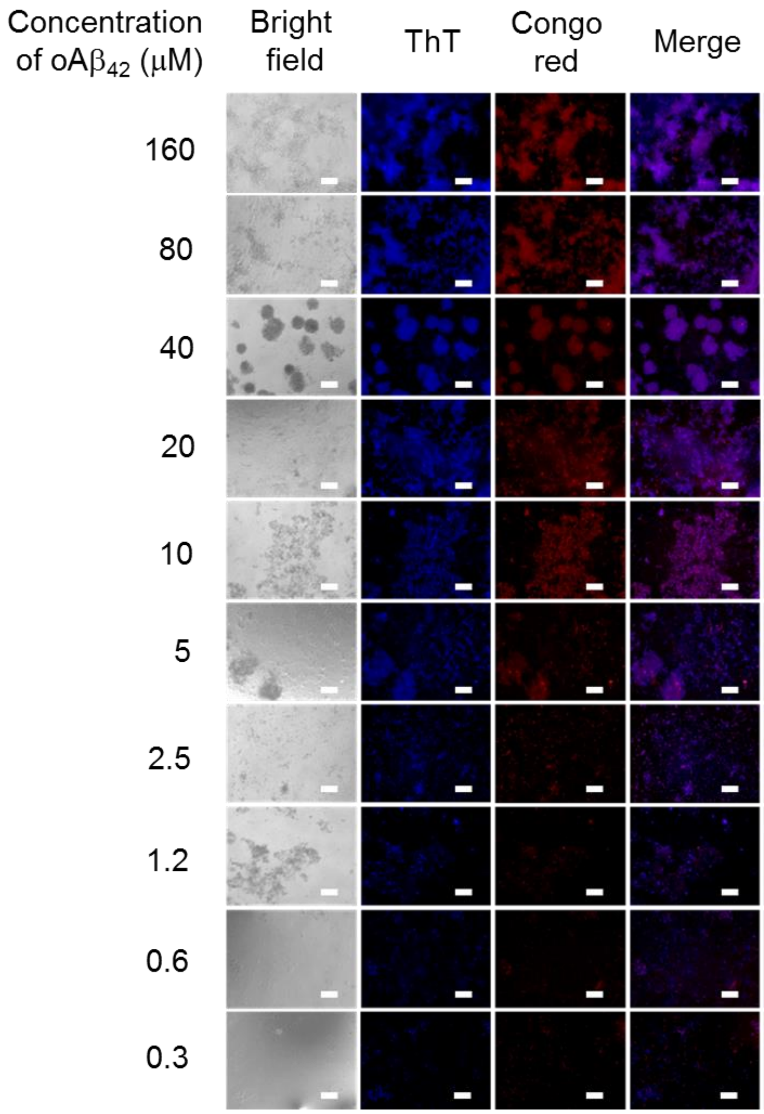

Fig. S4 Pronounced effect of capturing $oA\beta_{42}$ by magnetic stirring (2,500 rpm) with MSBs (144 μg/mL) into aggregates was observed by LSCM (n = 10). The $oA\beta_{42}$ was stained with ThT and CR. Scale bar: 100 μm.

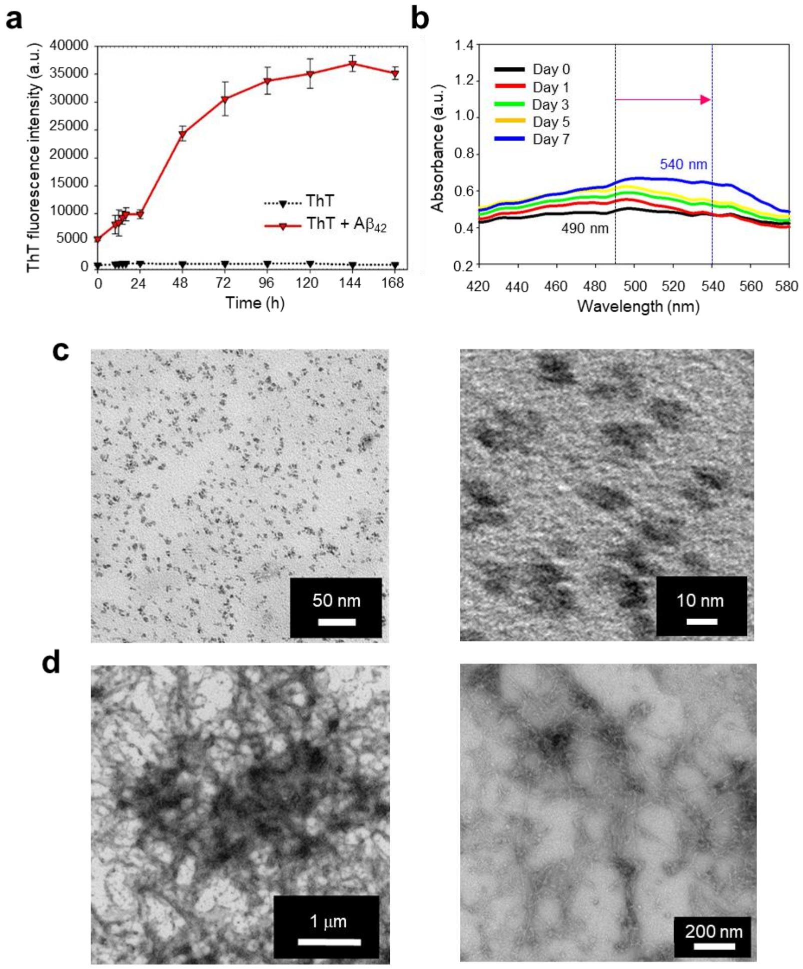

Fig. S5 Detailed structural characterizations of npAβ_{42}. (**a**) Fluorescence intensity of ThT in aqueous solution of Aβ_{42} (20 μM) as a function of time. (**b**) CR absorption spectra (420-580 nm) of Aβ_{42} (20 μM) with different incubation time intervals. (**c**) TEM and HR-TEM images of oAβ_{42}. (**d**) TEM and HR-TEM images of npAβ_{42}.

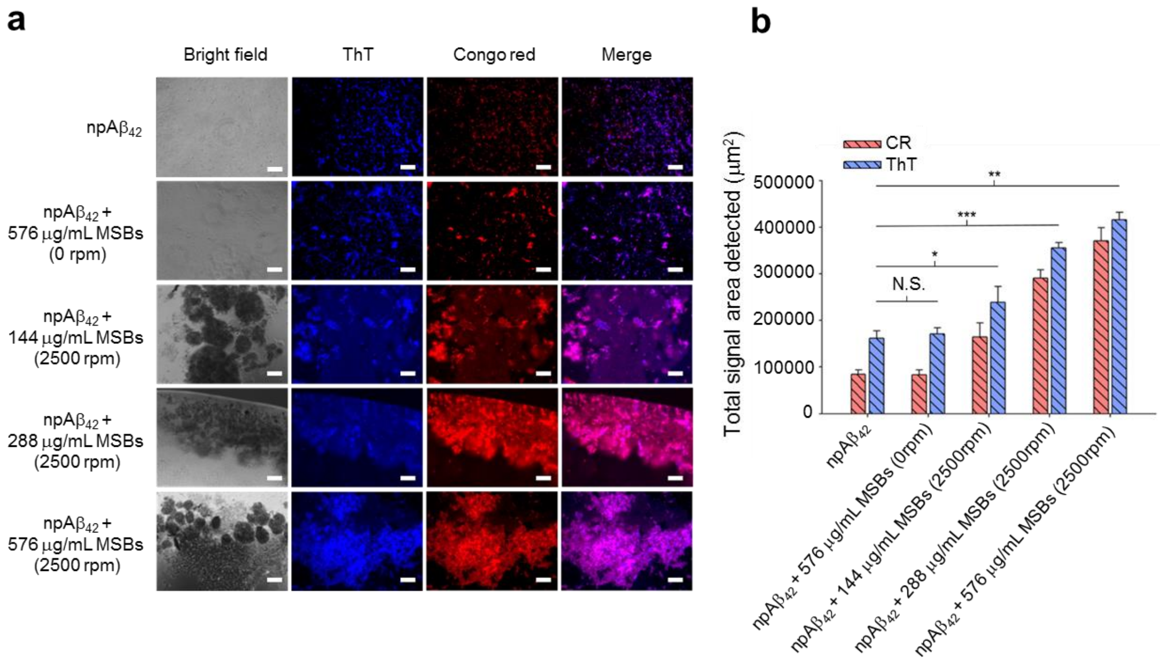

Fig. S6 Capture of np$A\beta_{42}$ into large aggregates by MSB stirring (n = 10). (**a**) Fluorescence images of np$A\beta_{42}$ treated with magnetic stirring as a function of the concentration of MSBs at 2,500 rpm for 20 min. The $A\beta_{42}$ concentration was 20 μM. The np$A\beta_{42}$ was attained from incubation of o$A\beta_{42}$ in PBS under mild shaking at 37°C for 7 days. Scale bar: 100 μm. (**b**) Total signal areas of large aggregates by ThT and CR staining. ***P < 0.005, **P < 0.01, *P < 0.05 and N.S. P > 0.05. Error bars represent mean ± s.d. (n = 10).

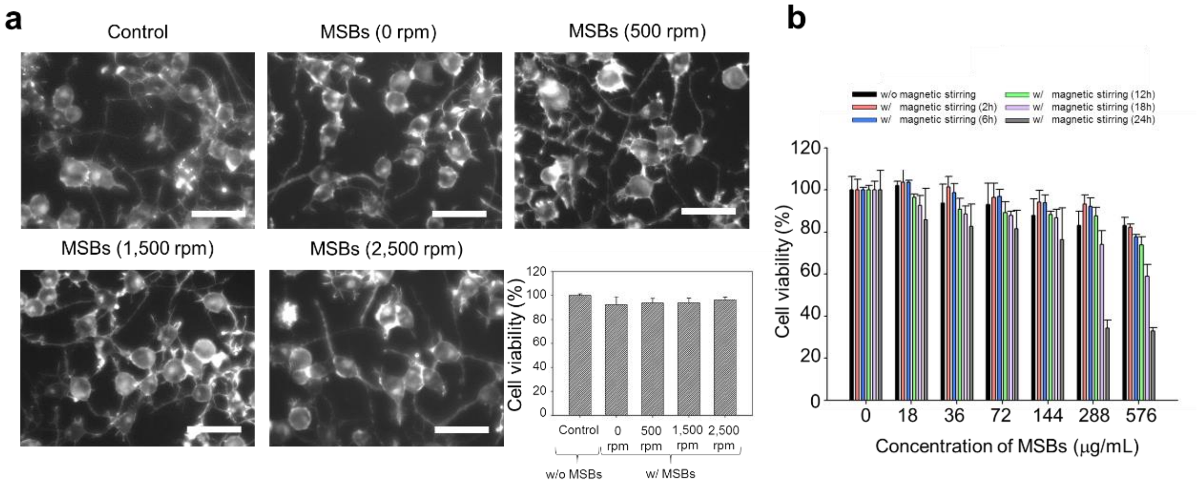

Fig. S7 (**a**) LSCM images of the neuron (N2a) outgrowth with magnetic stirring of various speeds for 2 h (MSB 144 μg mL^{-1}). Cytoskeleton was stained with F-actin marker. Scale bar: 100 μm. The viability of N2a cells receiving the magnetic stirring treatment with MSBs (144 μg mL^{-1}) at various stirring speeds. (**b**) Cell viability of N2a cells after the capture of oAβ_{42} by magnetic stirring with MSBs of different concentrations at 2,500 rpm for preset time intervals. The cell viability was evaluated by MTT assay. Error bars represent mean ± s.d. (n = 6).

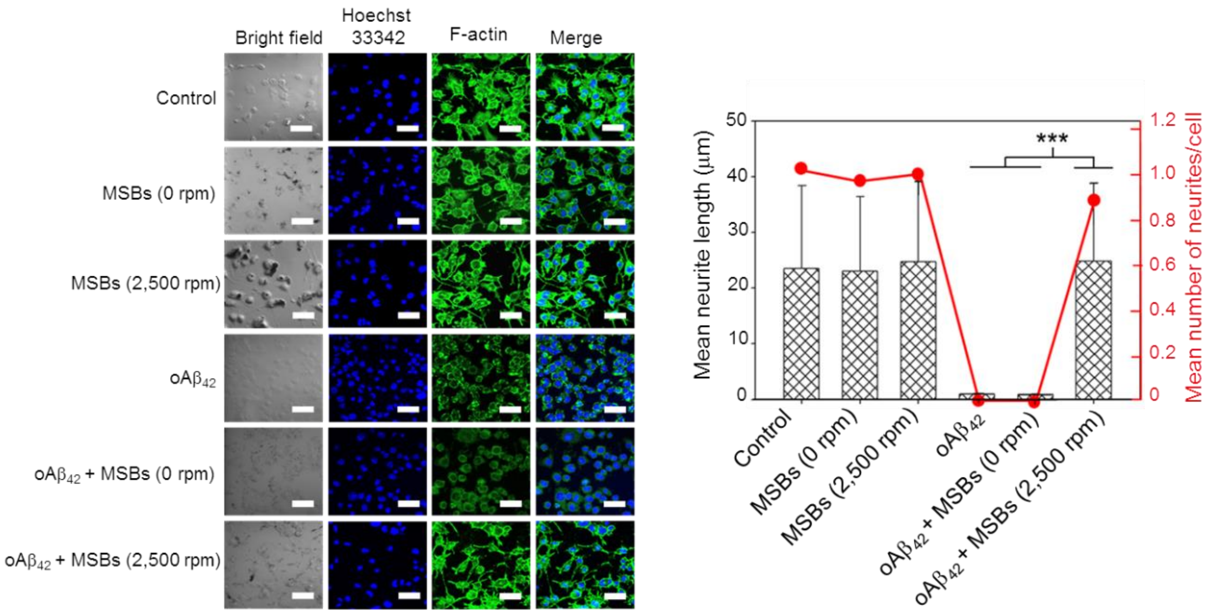

Fig. S8 Representative LSCM images of N2a cells before and after the magnetic stirring treatment. LSCM images of N2a cells treated with either $oA\beta_{42}$ only or $oA\beta_{42}$/MSBs ($oA\beta_{42}$ concentration: 160 μM; MSB 144 μg mL^{-1}) with and without magnetic stirring (2,500 rpm) for 2 h (n = 6). The cell nuclei and cytoskeleton were stained with Hoechst 33342 and F-actin marker, respectively. Scale bar: 50 μm. Quantitative data of neurite length (μm) and number with and without magnetic stirring treatment are also included. ***$P < 0.005$. Error bars represent mean ± s.d. (n = 6).

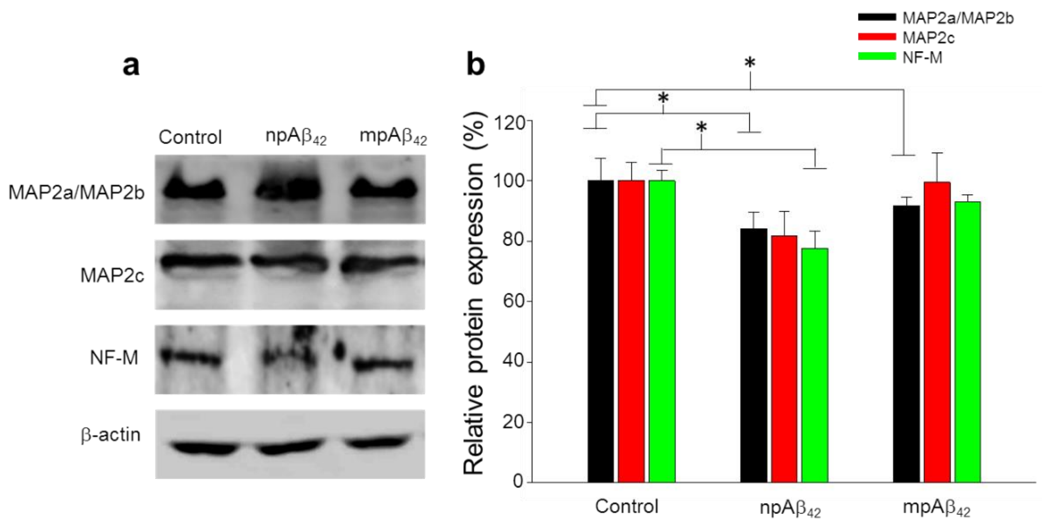

Fig. S9 Neuron-specific protein expression of N2a cells treated either with $npA\beta_{42}$ or $mpA\beta_{42}$ (n = 3). (**a**) Effects of $npA\beta_{42}$ and $mpA\beta_{42}$ on neuron-specific protein expression (MAP2a/MAP2b, MAP2c, NF-M and NeuN). β-actin was used as the loading control. (**b**) Relative signal intensities of individual neuron-specific proteins from N2a cells after the $npA\beta_{42}$ or $mpA\beta_{42}$ treatment. The $A\beta_{42}$ concentration was 160 μM. *$P < 0.05$. Error bars represent mean ± s.d. (n = 3).

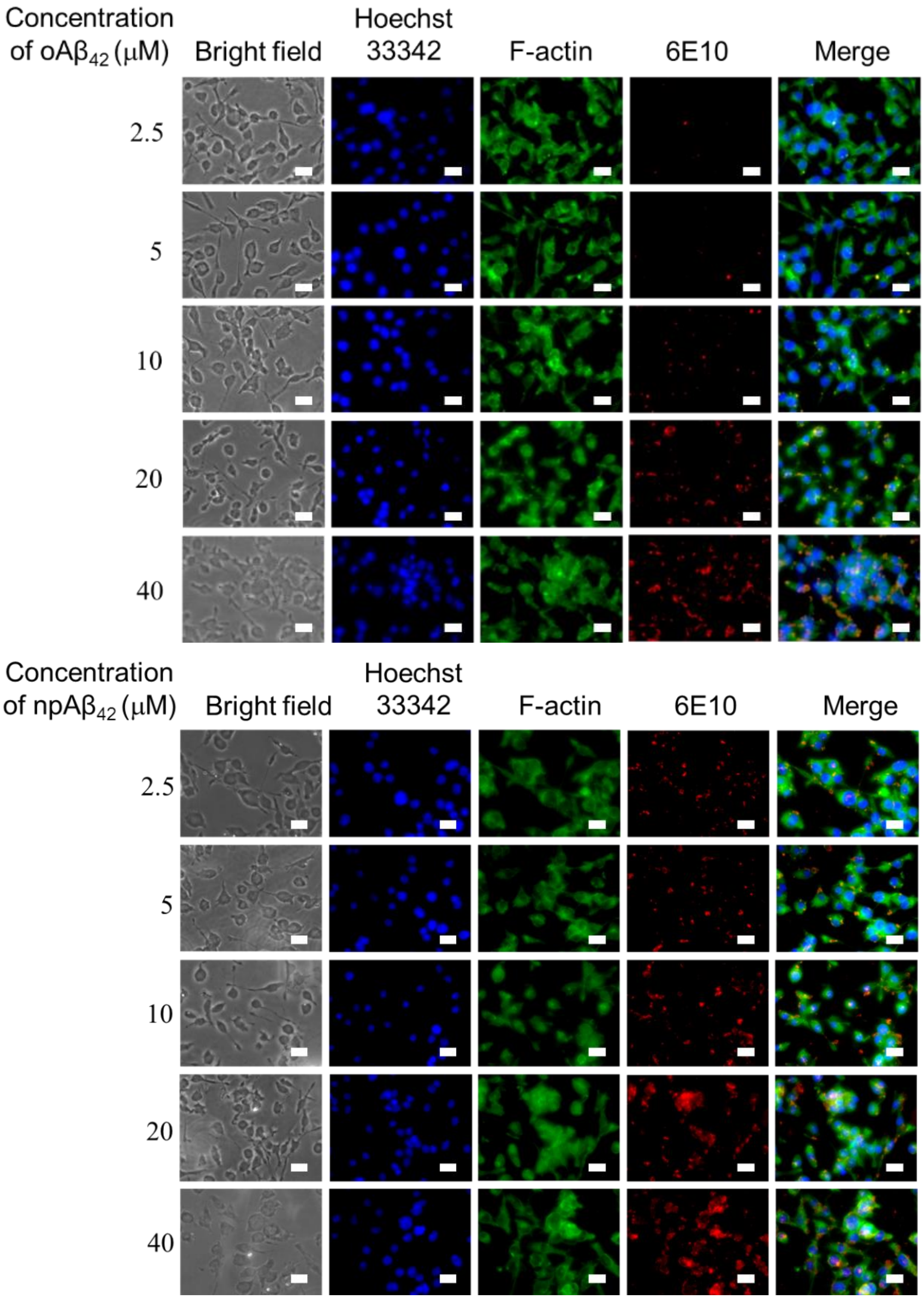

Concentration of oAβ42 (μM)
Bright field
Hoechst 33342
F-actin
6E10
Merge
2.5
5
10
20
40
Concentration of npAβ42 (μM)
Bright field
Hoechst 33342
F-actin
6E10
Merge
2.5
5
10
20
40

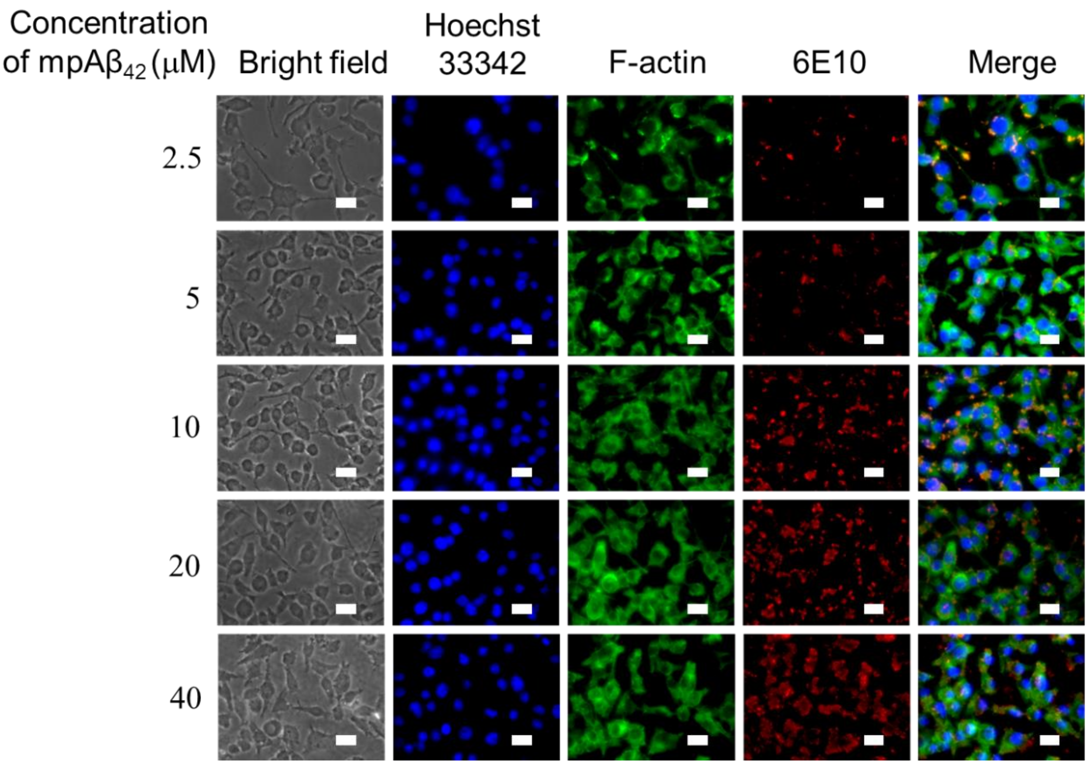

Fig. S10 Phagocytic actions of BV-2 cells toward oAβ_{42}, npAβ_{42} and mpAβ_{42}. Representative LSCM images of the Aβ_{42} uptake by BV-2 cells (n = 8). Aβ_{42} in different forms was IHC-stained using 6E10 as the primary antibody (λ_{ex} = 565 nm, λ_{em} = 680-730 nm). Cell nuclei and cytoskeleton were stained with Hoechst 33342 and F-actin marker, respectively. Scale bar: 50 μm.

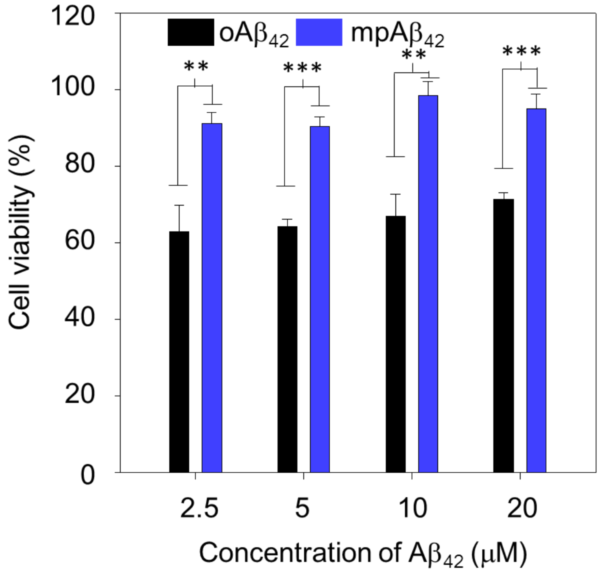

Fig. S11 Cell viability of N2a cells after 24 h-incubation in conditioned media collected separately from the co-incubations of BV-2 cells with $oA\beta_{42}$ and BV-2 cells with $mpA\beta_{42}$. ***P < 0.005, **P < 0.01. Error bars represent mean ± s.d. (n = 6).

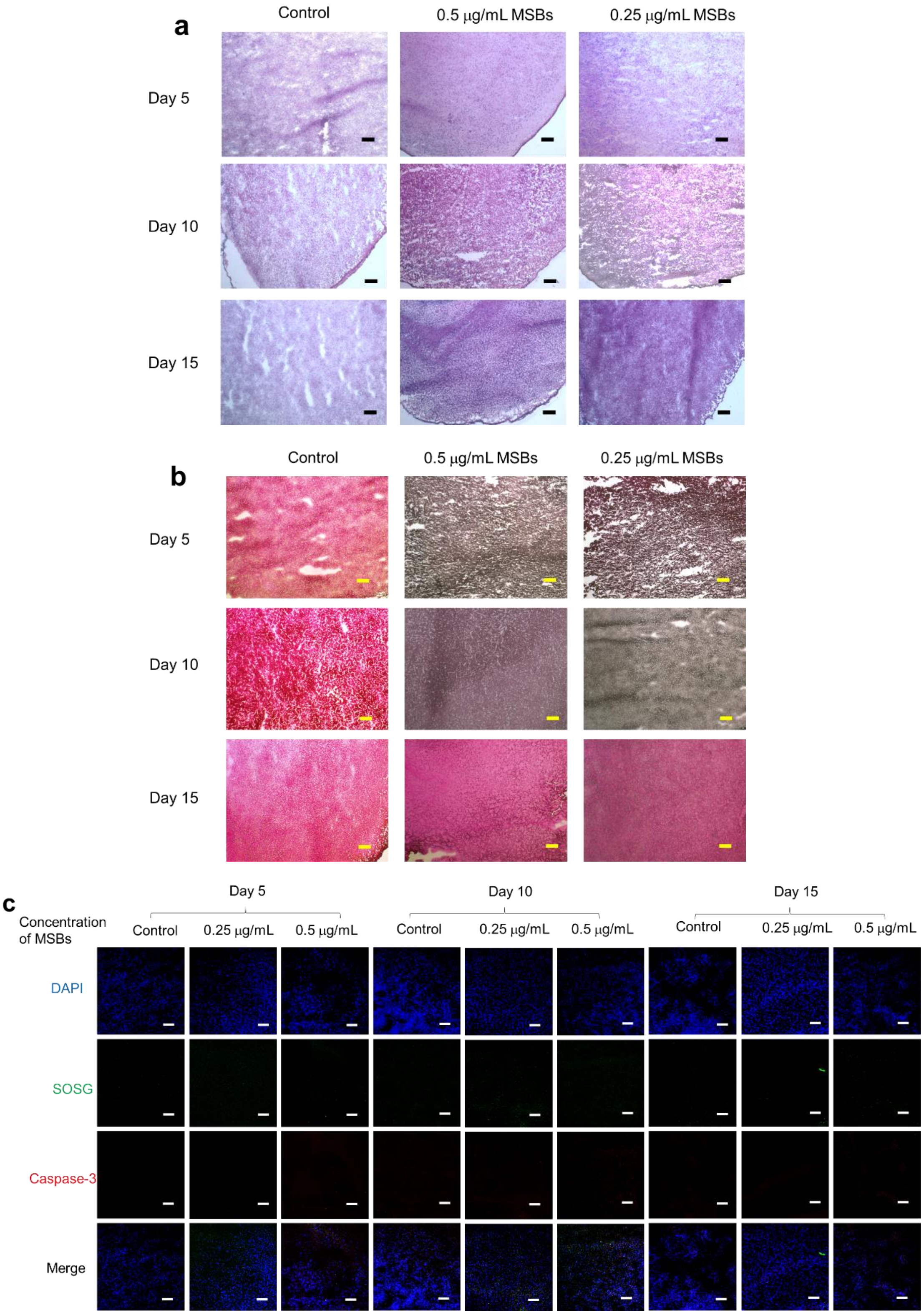

Fig. S12 Brain tissue examination with (**a**) H&E (**b**) prussian blue (MSBs) and (**c**) DAPI (nuclei), SOSG (ROS) and caspase-3 (cell apoptosis) IHC staining from healthy C57BL/6J mice receiving MSB stirring treatment (2,400 rpm, 20 min) (n = 3). Scale bars for (**a**) and (**b**): 500 μm. Scale bar for (**c**): 100 μm.

Supplementary Table

Table S1 Characterization of OA-SPIONs and LA/OA-SPIONs by DLS

Sample	D_h (nm)[a]	PDI[b]	ζ-potential (mV)
OA-SPIONs	34±2	0.15±0.01	-
LA/OA-SPIONs	40±2	0.10±0.01	-34.4±0.4

[a]Mean hydrodynamic particle diameters of OA-SPIONs in hexane and LA/OA-SPIONs in PBS. [b]Polydispersity index.
